# Essential role of diastolic oscillatory potentials in adrenergic control of guinea pig sino-atrial node discharge

**DOI:** 10.1186/1423-0127-16-101

**Published:** 2009-11-18

**Authors:** Mario Vassalle, John N Catanzaro, Michael P Nett, Marcello Rota

**Affiliations:** 1 Department of Physiology and Pharmacology, Box 31, State University of New York, Downstate Medical Center, 450 Clarkson Avenue, Brooklyn, NY 11203, USA; 2 Department of Cardiology, North Shore University Hospital, 300 Community Drive, Manhasset, New York 11030, USA; 3 Current address: Insall Scott Kelly Institute for Orthopedics and Sports Medicine, 210 East 64th Street, Fourth floor, New York, NY 10065, USA; 4 Current address: Harvard Medical School, Brigham & Women's Hospital, Departments of Anesthesia and Medicine, 75 Francis St, Thorn Building, Room 1228A, Boston, MA 02115, USA

## Abstract

**Background:**

The diastolic oscillatory after-potential V_os _and pre-potential ThV_os _play an essential role in the pacemaker mechanism of sino-atrial node (SAN). The aim of this study was to investigate whether these oscillatory potentials are also involved in adrenergic control of SAN discharge.

**Methods:**

V_os _and ThV_os _were visualized by superfusing guinea pig SAN in high [K^+^]_o_. The actions of adrenergic agonists on oscillatory potentials were studied by means of a microelectrode technique. Statistical significance was determined by means of Student's paired *t*-test.

**Results:**

In non-spontaneous SAN, norepinephrine (NE) decreased the resting potential into a voltage range ("oscillatory zone") where increasingly larger ThV_os _appeared and initiated spontaneous discharge. In slowly discharging SAN, NE gradually increased the rate by increasing the amplitude and slope of earlier-occurring ThV_os _and of V_os _until these oscillations fused with initial diastolic depolarization (DD_1_). In the presence of NE, sudden fast rhythms were initiated by large V_os _that entered a more negative oscillatory zone and initiated a large ThV_os_. Recovery from NE exposure involved the converse changes. The β-adrenergic agonist isoproterenol had similar actions. Increasing calcium load by decreasing high [K^+^]_o_, by fast drive or by recovery in Tyrode solution led to growth of V_os _and ThV_os _which abruptly fused when a fast sudden rhythm was induced. Low [Ca^2+^]_o _antagonized the adrenergic actions. Cesium (a blocker of I_f_) induced spontaneous discharge in quiescent SAN through ThV_os_. In spontaneous SAN, Cs^+^increased V_os _and ThV_os_, thereby increasing the rate. Cs^+ ^did not hinder the positive chronotropic action of NE. Barium increased the rate, as Cs^+ ^did.

**Conclusion:**

Adrenergic agonists: (i) initiate SAN discharge by decreasing the resting potential and inducing ThV_os_; (ii) gradually accelerate SAN rate by predominantly increasing size and slope of earlier and more negative ThV_os_; (iii) can induce sudden fast rhythms through the abrupt fusion of large V_os _with large ThV_os_; (iv) increase V_os _and ThV_os_by increasing cellular calcium; and (v) do not modify the oscillatory potentials by means of the hyperpolarization-activated current I_f_. The results provide evidence for novel mechanisms by which the SAN dominant pacemaker activity is initiated and enhanced by adrenergic agonists.

## Background

The mechanisms by which adrenergic agonists increase the rate of the sino-atrial node (SAN) are not agreed upon [1-3]. In part, this is the result of the disagreement about the SAN dominant pacemaker mechanism [see [4,5]], since adrenergic neuromediators would be expected to change SAN rate by acting on dominant pacemakers.

Adrenergic neuromediators affect several voltage- and time-dependent currents purported to be involved in SAN dominant pacemaker mechanism, including the calcium current I_CaL _[6-8], the delayed rectifier current I_K _[1,9,10] and the hyperpolarization-activated current I_f _[2].

In addition, the Na^+^-Ca^2+ ^exchange current (I_Na-Ca_) contributes to the pacemaker activity in the absence and presence of adrenergic agonists [11,12]. Stimulation of β-adrenergic receptors increases I_Ca _[6], the Ca^2+ ^transient [8,13] and the uptake and release of Ca^2+ ^by the sarcoplasmic reticulum (SR) [3,12]. A larger Ca^2+ ^release during late diastole increases I_Na-Ca _and therefore the rate of discharge [8,11,14].

This complex state of affairs is complicated by the fact that (in addition to diastolic depolarization, DD) two oscillatory potentials (the after-potential V_os _and the pre-potential ThV_os_) are obligatory components of SAN dominant pacemaker mechanism [15-18]. In Tyrode solution, as soon as the maximum diastolic potential (MDP) of SAN dominant pacemakers is reached, the membrane potential turns around into a DD whose slope is similar to that of the preceding final phase 3 repolarization. This U-shaped DD merges smoothly into the slow upstroke of the action potential (AP).

When [K^+^]_o _is suitably increased, all APs of SAN display a slow upstroke, a less negative MDP and a U-shaped DD [15-19], as dominant pacemakers do in Tyrode solution. On further increase of high [K^+^]_o_, the threshold for the upstroke is missed and the oscillatory potentials V_os _and ThV_os _become unmasked. V_os _is obligatorily superimposed on the initial diastolic depolarization (DD_1_) whereas ThV_os _appears gradually later when the late DD (DD_2_) enters a less negative voltage range ("oscillatory zone"). With sufficiently high [K^+^]_o_, only a small DD is left and quiescence follows [17]. Quiescence is due to the fact that high [K^+^]_o _decreases the oscillatory potentials by decreasing intracellular calcium through an enhanced extrusion of calcium by the Na^+^-Ca^2+ ^exchange (see 17) and also by decreasing I_Ca _[20]. The converse changes occur when high [K^+^]_o _is decreased to normal value.

Our aim was to test the hypothesis that adrenergic agonists increase the SAN rate also by modifying the oscillatory potentials. This hypothesis is based on the fact that, on the one hand, V_os _and ThV_os _are increased by a greater Ca^2+ ^loading [15-19] and, on the other hand, adrenergic agonists increase cellular Ca^2+ ^[6,13,21].

The results obtained show that adrenergic agonists initiate spontaneous discharge and increase SAN rate (gradually or suddenly) by modifying in different ways V_os _and ThV_os _as well as the resting potential and DD.

## Methods

The experiments conform to the principles of national and international ethical guidelines. The experimental protocols were approved by the local Animal Care and Use Committee.

The Methods have been reported in detail [15-18]. In brief outline, eighteen Hartley adult guinea pigs of either sex (weighing 610 ± 59 gr) were euthanized with sodium pentobarbital (60 mg/kg, intraperitoneally). Once the respiration had stopped, the heart was rapidly excised and placed in a Petri dish filled with oxygenated Tyrode solution.

The sino-atrial node was separated from the surrounding red-brown atrial tissue. The SAN was superfused at 37°C with oxygenated (95% O_2 _and 5% CO_2_) Tyrode solution of the following composition (mM): NaCl 129, KCl 4, CaCl_2 _2.7, NaHCO_3 _20, NaH_2_PO_4 _0.45, MgCl_2 _1.05 and glucose 5.5. Membrane potentials were recorded by means of glass microelectrodes filled with 3 M KCl and coupled to a Dagan probe and to a Dagan model 8500 operational amplifier. Contractile force was recorded by means of a force transducer (Grass Model FTO3C) connected to a Grass Model 7D Polygraph. The traces were displayed on a Tektronix model 5111 storage oscilloscope and recorded on paper on a 3-channel chart recorder (Gould Brush 2400).

To analyze the characteristics of V_os _(also referred to in the literature as oscillatory afterpotential or delayed afterdepolarization) and of ThV_os _(the oscillatory potential leading to the threshold for the upstroke [22]), [K^+^]_o _was increased by mixing the Tyrode solution with a solution containing 40 mM KCl, but otherwise identical with Tyrode solution and gassed with the same mixture. The flow of Tyrode solution was kept constant (Gilson pump, model PP-4-A) and the flow of the K^+^-rich solution was changed by means of a peristaltic pump (LKB, 12000 Varioperpex). The [K^+^]_o _of the final admixture was calculated by means of an equation, as detailed previously [15-18]. The effects of a higher [K^+^]_o _were quickly established (<5 min): once a new steady-state was attained, tests were carried out as warranted. The use of the guinea-pig isolated SAN permitted to abolish the electrotonic interference from atrial muscle, since the SAN is made of sinus cells throughout its thickness [23].

Exposure to suitably high [K^+^]_o _made the SAN electrically more homogeneous, since (as a consequence of depolarization) the APs assumed a dominant configuration (smooth transition from U-shaped DD into a slow upstroke and smaller APs). Eventually, high [K^+^]_o _permitted the separation of V_os_, ThV_os _and DD [15-18]. That the cells studied in high [K^+^]_o _were pacemaker cells is shown by the persistence of their DD during recovery in Tyrode solution. Thus, the cells studied were pacemaker cells with dominant characteristics. Secondary pacemakers have a more negative diastolic potential, abrupt transition between diastolic potential and upstroke, much larger AP, less steep DD and no ThV_os_, since the more negative diastolic potential is negative to the oscillatory zone [e.g., [19]].

The abbreviations V_os _and ThV_os _are used for both singular and plural. The early diastolic depolarization is referred to as DD_1 _and the late diastolic depolarization as DD_2_. The presence and amplitude of V_os _was identified by its peak during DD_1_. The maximum diastolic potential (MDP) and the resting potential are said to decrease when becoming less negative. When in control the SAN was quiescent in high [K^+^]_o_, the characteristics of AP, V_os _and ThV_os _were measured just before stoppage in order to determine their modifications by the subsequent interventions.

Norepinephrine bitartrate (NE), isoproterenol hydrochloride, cesium chloride and barium chloride (Sigma) were added to the Tyrode solution and the final concentration in the bath was calculated by taking into account the flow of high [K^+^]_o _solution. The results of the tests performed (*n*) are expressed as means ± standard error of the mean (SEM). Student's paired *t*-test was used and a P value < 0.05 was considered significant (indicated in text and figures by an asterisk, *).

## Results

### Initiation of spontaneous discharge by norepinephrine

**Figure 1:**
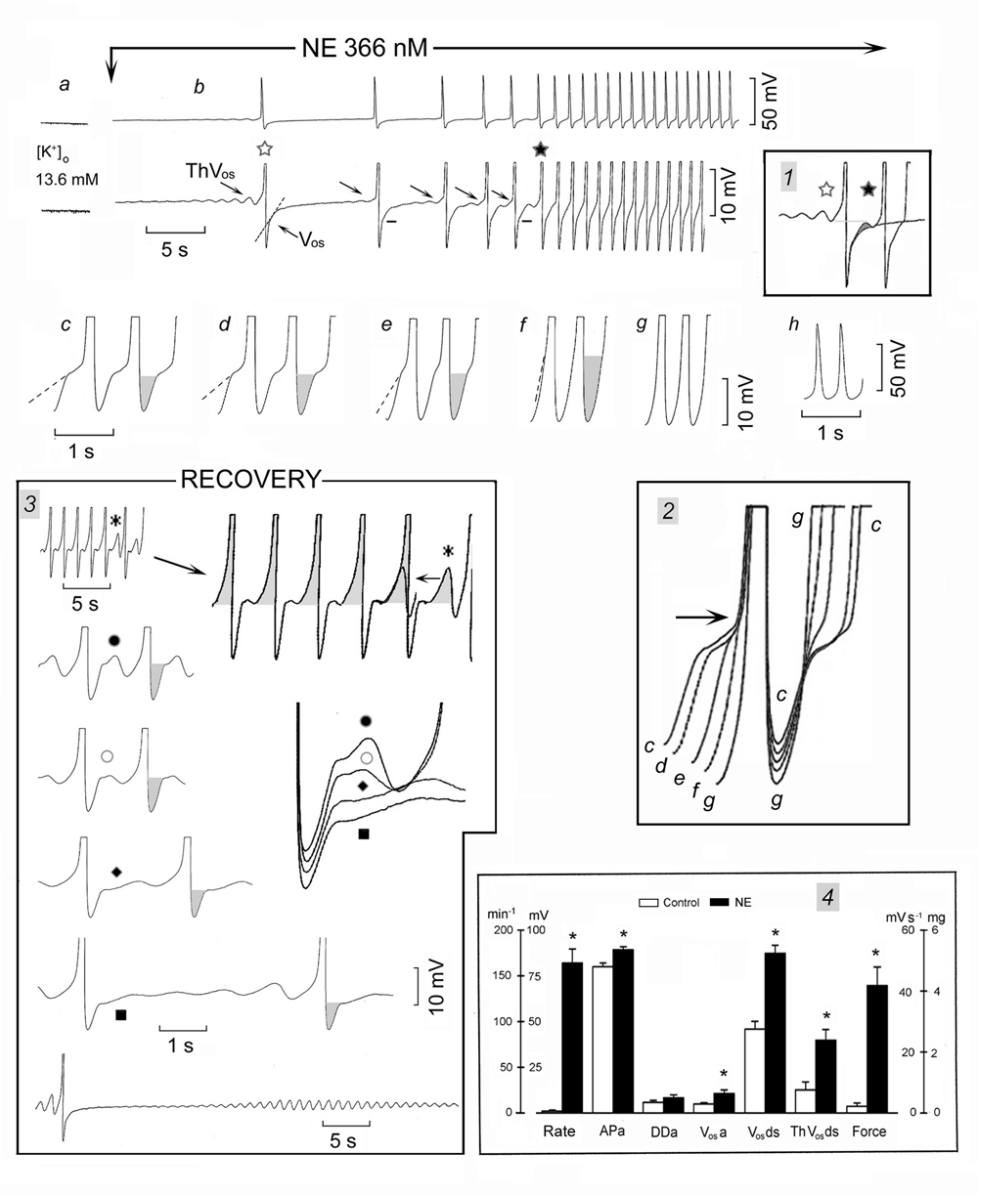
**Norepinephrine induces SAN discharge through ThV_os_**. In 13.6 mM [K^+^]_o_, NE was administered during the recording of panels *b-h *at normal and at higher gain. In panel *b*, the rightward oblique arrows indicate ThV_os _and the leftward oblique arrow V_os_. The dash lines emphasize the change in slope of DD_1 _past the peak of V_os_, the peak being indicated by the short bars next to DD_1_. The traces labeled with an empty and a filled star were superimposed in inset *1*. In the higher gain *c*-*f *traces, the shaded areas emphasize the progressive growth of V_os_. The *c-g *traces have been superimposed in inset *2*, the arrow pointing to the shortening of the AP. The events during the recovery from NE exposure are illustrated in inset *3*. The top trace was recorded also at higher gain (see rightward oblique arrow) and the ThV_os _marked by an asterisk has been superimposed upon the previous AP (horizontal arrow). In panel *4*, means and standard error of the mean are shown in control (empty columns) and in the presence of NE (filled columns). The parameters shown are the rate in min^-1^, the action potential amplitude in mV (APa), the diastolic depolarization amplitude in mV (DDa), the amplitude of V_os _in mV, the depolarizing slope of V_os_(V_os_ds) and of ThV_os _(ThV_os_ds) in mV s^-1^, and force in mg. Asterisks indicate statistical difference with respect to control (P < 0.05).

In Fig. 1, the SAN was quiescent in high [K^+^]_o _(panel *a*) and NE decreased the resting potential and made ThV_os _appear (first rightward oblique arrow) that initiated an AP (panel *b*). The AP was followed by a V_os _(leftward oblique arrow). The dash line emphasizes the transition between the peak of V_os _and the subsequent DD. ThV_os _appeared progressively sooner (subsequent rightward oblique arrows) and at more negative potentials. The traces labeled with an empty and a filled star were superimposed in inset *1 *and show that NE made ThV_os _(shaded area) begin early in diastole and at a more negative value (negative shift of the oscillatory zone). By occurring earlier in diastole, ThV_os _gradually increased the rate. At the same time, V_os _size increased (short bars). After the AP labeled by a filled star, ThV_os _and V_os _became fused (see also inset *1*).

Subsequent changes are shown at greater time base in panels *c*-*g*. V_os _increased progressively, as emphasized by the shaded areas (panels *c-f*). After the peak of V_os_, the depolarizing phase (presumably of ThV_os_) became steeper (dash lines), until in panel *g *only a U-shaped DD was present. In inset *2*, the superimposed *c-g *traces show that NE shortened the AP (arrow), increased the MDP (from trace *c *to *g*), and steepened the slope of V_os _as well as of ThV_os_. As shown in panel *h *(recorded simultaneously with panel g), much larger APs discharged at 150 min^-1^.

During the recovery from NE exposure (inset *3*), the converse changes occurred. As shown by the shaded areas in the enlarged top trace (rightward oblique arrow), the slowing of discharge initially was due to a gradually less steep slope during later diastole. Eventually, ThV_os _missed the threshold for the upstroke (asterisk), as emphasized by its superimposition on the previous AP (indicated by the horizontal arrow). This indeed shows that the peak of V_os _was followed by the depolarizing slope of ThV_os_.

The traces labeled with dot, circle, rhombus and square show that, during the recovery from NE exposure, the amplitude of V_os _gradually decreased (shaded areas), the early ThV_os _decreased in amplitude and the subsequent ThV_os _attained the threshold gradually later. The superimposed traces (displaced vertically for clarity) emphasize the decrease in V_os _and ThV_os _amplitude, the gradual slowing being due to the later appearance of ThV_os _during DD_2_. In the bottom trace, the last AP was followed by ThV_os_, which increased in amplitude, failed to attain the threshold and progressively declined to the resting potential ("diamond pattern").

Thus, ThV_os _was responsible solely for the initiation of spontaneous discharge and predominantly for the gradual increase in rate. The earlier, larger and more negative ThV_os _eventually fused with the larger V_os _into the U-shaped DD.

In 12.3 ± 1.0 mM [K^+^]_o_, SAN was quiescent in *n *= 10 and slowly driven in *n *= 3 at an average 9 beats min^-1^. Norepinephrine (415 ± 77 nM) decreased the resting or diastolic potential by 2.7 ± 0.28* mV by the time it consistently induced ThV_os_, thereby initiating spontaneous discharge. As shown in Fig. 1, inset *4 *(means and SEM), NE increased the rate from 2.08 to 164 ± 15* min^-1^, the action potential amplitude (APa) 11%*, diastolic depolarization amplitude (DDa) 43%, V_os _amplitude (V_os_a) 112%*, V_os _depolarizing slope (V_os_ds) 55%*, ThV_os _depolarizing slope (ThV_os_ds) 219%* and force 1800%*.

### Increase in spontaneous rate of discharge by norepinephrine

Since the SAN is generally active, [K^+^]_o_, was increased to a level that permitted at the same time spontaneous discharge and the visualization of both V_os _and ThV_os_. In this way, it was possible to verify how NE progressively modified the oscillatory potentials while increasing the rate.

**Figure 2:**
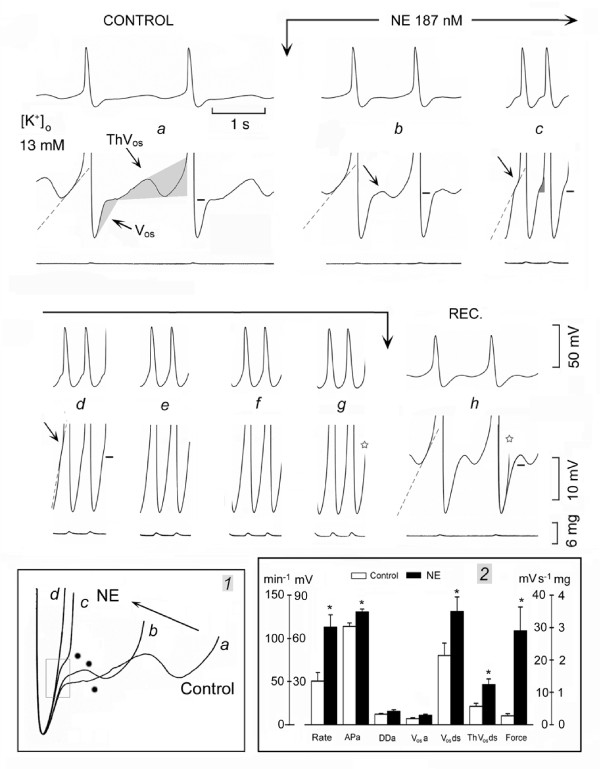
**Norepinephrine increases SAN discharge by increasing ThV_os _and V_os_**. The SAN was spontaneously active in 13 mM [K^+^]_o_. In panel *a*, the shaded areas emphasize V_os _and ThV_os_, as labeled. The dash lines extrapolate the depolarizing slope of ThV_os_. NE was administered between the vertical arrows (*b*-*g *panels). Inset *1 *shows the gradual increase of ThV_os _(dots) and V_os _(see rectangle) by NE. In inset *2*, means and SEM in control and in the presence of NE are shown. Other explanations are as in the legend of Fig.1.

In Fig. 2A, in control (panel *a*), the SAN was discharging at 30.7 beats min^-1 ^and the AP was followed by a V_os _(leftward oblique arrow), whose peak is indicated by the short bar. In turn, V_os _was followed by a sub-threshold ThV_os _(rightward oblique arrow). The depolarizing phase of the subsequent ThV_os _was faster and larger, and attained the threshold for the upstroke (end of shaded area). NE increased V_os _amplitude (upward shift of V_os _peak, marked by short bars), caused ThV_os _to appear much earlier during DD_2 _(rightward oblique arrows in panels *b*, *c *and *d*), and increased the slope of ThV_os _depolarizing phase (dash lines, panels *b-d*).

As a consequence of the larger V_os _and of the earlier onset and steeper slope of ThV_os _(small shaded area in panel *c*), the threshold for the upstroke was attained much sooner. In panels *e-g*, the inflection was no longer clearly visible as V_os _and ThV_os _were fused into the U-shaped DD. The rate increased to 166 min^-1 ^(panel *g*, 440% of control).

In inset *1*, the superimposed traces show that NE increased the rate (leftward oblique arrow) by gradually increasing the slope and amplitude of earlier ThV_os _(dots) and of V_os _(rectangle). During partial recovery (panel *h*), slowing of the rate was due to the first ThV_os _missing the threshold and to the decrease toward control value of depolarizing slope of the second ThV_os _(dash line). The depolarizing phase of V_os _was also slower, as shown by the superimposition of the trace labeled with empty star in panel *g *on the recovery trace in panel *h*.

In *n *= 14, the SAN was spontaneously active in 15.1 ± 1.8 mM [K^+^]_o_. As shown in inset *2*, NE (469 ± 55 nM) changed the rate by 124%*, APa 15%*, DDa 30%, V_os_a 54%*, V_os_ds 64%*, ThV_os_ds 120%* and force 974%*. Thus, NE increases the rate of spontaneously discharging SAN by shifting the diastolic potential in a positive direction, and by increasing the amplitude and slope of already present V_os _and ThV_os_. Also, NE increased the amplitude and decreased the duration of AP (-13.3%*), and increased force, as it does in Tyrode solution.

### Sudden initiation of fast discharge by adrenergic activation

*In vivo*, sympathetic nerve stimulation induces a gradual increase in the rate of SAN and (in complete atrio-ventricular block) of the idioventricular rhythm originating in Purkinje fibers. However, adrenergic activation may also induce a sudden marked acceleration of the idioventricular rhythm [24].

**Figure 3:**
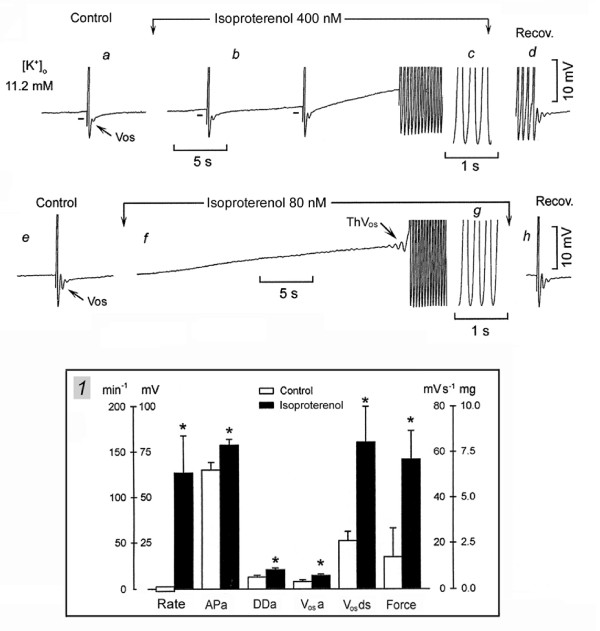
**Sudden onset of fast discharge in the presence of isoproterenol**. In panel *a*, the non-spontaneous SAN was driven at 6 min^-1 ^in 11.2 mM [K^+^]_o_. Isoproterenol was administered between the downward arrows. In panel *b*, the first 3 APs were driven, but once the fast sudden rhythm started, the drive was discontinued. The sudden cessation of the fast rhythm is shown in panel *d*. In panel *e*, the same SAN was driven at 6 min^-1^, but the drive was discontinued prior to isoproterenol administration. In panel *f*, the arrow points to the ThV_os _that initiated the sudden fast rhythm. The fast rhythm ceased during recovery and a driven AP is shown in panel *h*. The means and SEM are shown in inset *1*. Other explanations are as in the legend of Fig. 1.

Adrenergic agonists could suddenly induce a fast rhythm also in SAN superfused *in vitro *and therefore allow the study of the underlying mechanisms. In Fig. 3, the non-spontaneous SAN was driven in high [K^+^]_o _at 6 min^-1 ^and the AP was followed by a V_os_, but no ThV_os _were present (panel *a*). In panel *b*, adding the β-adrenergic agonist isoproterenol decreased the diastolic potential and increased the amplitude of V_os _(short bars). The third driven AP initiated a sudden rhythm at 240 min^-1^. The drive was stopped and the fast rhythm continued as long as isoproterenol was superfused. During recovery in high [K^+^]_o _(panel *d*), the brief slowing and sudden stoppage was due to the late DD_1 _slope becoming slower and suddenly missing the threshold. Damped oscillations followed.

In Fig. 3e, in control, the driven AP was followed by V_os _whose peak barely reached the resting potential. The drive was stopped and isoproterenol administration decreased the resting membrane by ~7 mV. ThV_os _appeared (arrow) which elicited an AP that initiated a sudden fast rhythm (254 min^-1 ^in panel *g*). In panel *h*, during the recovery from isoproterenol exposure, the driven AP was again followed by large damped oscillations.

In inset *1*, *n *= 6, in 12.6 ± 1.4 mM [K^+^]_o_, the SAN was not spontaneously active and was either driven at 6 min^-1^ or left quiescent. Isoproterenol (114 ± 53 nM) decreased the resting or diastolic potential by 4.4 ± 0.9* mV and increased the spontaneous rate from zero to 127 ± 40* min^-1^, APa 22%*, DDa 59%*, V_os_a 78%*, V_os_ds 209%* and force 313%*. The effects were similar to those induced by NE, thus indicating that they are related to stimulation of β receptors.

In *n *= 9, in 10.8 ± 0.5 mM [K^+^]_o_, the SAN was quiescent. NE or isoproterenol (mean value 520 ± 0.13 nM) induced a sudden fast rhythm (214 ± 14* min^-1^) either with the first AP or after a few accelerating APs. In *n *= 22, in 13.3 ± 1.5 mM [K^+^]_o_, the SAN was either quiescent or spontaneously active (mean rate 17 beats min^-1^). NE or isoproterenol (mean value 459 ± 170 nM) induced or increased ThV_os _that resulted in a gradual acceleration to 105 ± 9* min^-1^. In 6/22, the gradual increase led to a sudden acceleration to 190 ± 17 min^-1 ^(* with respect to the gradually increased rhythm).

### Role of V_os _and ThV_os _in sudden fast rhythms on decreasing high [K^+^]_o_

High [K^+^]_o _was decreased (to increase [Ca^2+^]_i_) in order to investigate whether and how V_os _and ThV_os _induce a sudden fast rhythm, as adrenergic agonists do.

**Figure 4:**
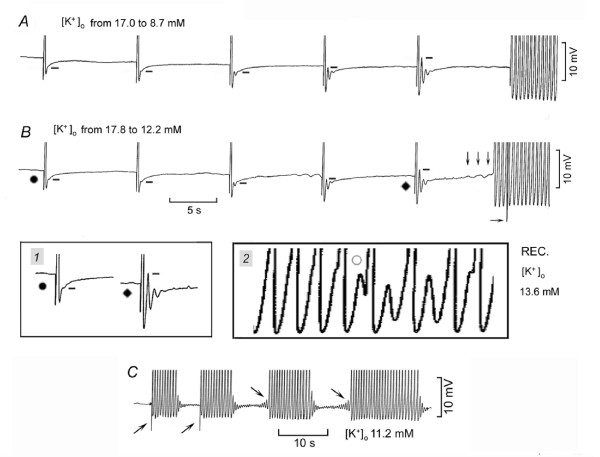
**Induction of sudden fast rhythm by V_os _and ThV_os _on lowering high [K^+^]_o_**. In panels *A *and *B*, [K^+^]_o _was decreased while the same SAN was driven. The bars mark the peak of V_os_. The downward arrows mark the ThV_os _that initiated an AP followed by the fast rhythm. The horizontal arrow points to the stimulus artifact of the last driven AP. The traces labeled with a dot and a rhombus are shown at higher gain in inset *1*. In inset *2*, the circle labels a ThV_os _that missed the threshold during the subsiding of the fast rhythm. In panel *C*, the upward oblique arrows indicate driven APs that initiated runs of fast discharge. The downward oblique arrows indicate ThV_os _that initiated an AP followed by runs of fast discharge.

In Fig. 4A, [K^+^]_o _was decreased while the SAN was driven at 6 min^-1^. The resting potential became more negative and V_os _(whose peak is marked by short bars) gradually increased until the peak of V_os _actually overshot the resting potential. Small ThV_os _appeared, but it was the last driven AP that initiated a sudden fast rhythm (146 min^-1^). During recovery, when the threshold was missed, diastolic oscillations were unmasked (not shown).

In Fig. 4B (same SAN), again [K^+^]_o _was decreased while driving the SAN at 6 min^-1 ^As in Fig. 4A, the APs were followed by V_os _which gradually increased in size (see traces labeled by a dot and a rhombus without and within inset *1*). However, this time larger ThV_os _(downward arrows) elicited an AP that initiated a sudden fast rhythm ahead of the next driven AP (horizontal arrow). As shown in inset *2*, during the recovery in higher [K^+^]_o_, the U-shaped DD began to miss the threshold (circle), thereby unmasking large diastolic oscillations. The oscillations appear to include ThV_os_, since they undershot DD, a hyperpolarization typical of ThV_os _but not of V_os _[17,18]. Thus, on lowering [K^+^]_o_, V_os _increased progressively and a fast rhythm could follow either a driven or a ThV_os_-induced AP, as in the case of adrenergic agonists.

In Fig. 4C, in the same SAN, driven APs (rightward oblique arrows) initiated trains of fast rhythm. The termination of the fast rhythm unmasked large damped oscillations. After the first two trains, drive was stopped and during the subsequent longer DD_2_, increasingly larger ThV_os _appeared (downward oblique arrows), which also initiated trains of fast rhythm. The termination of these trains was followed by damped oscillations, which showed large hyperpolarizing phases, typical of ThV_os_. Thus, the growth of both V_os _and ThV_os _might be involved in the induction of sudden fast rhythms by APs elicited by drive or by ThV_os_.

When [K^+^]_o _was decreased from a higher value to 11.2 ± 0.4 mM, the SAN was either quiescent (*n *= 9) or driven at 6 min^-1 ^(*n *= 11). ThV_os _initiated a fast rhythm in all the quiescent preparations and in 3 of the driven preparations. In the remainder, the fast rhythm was initiated by driven APs. The rate the sudden rhythm was 130 ± 3.7* min^-1^. When the fast rhythm subsided, large oscillations were unmasked.

### Role of V_os _and ThV_os _in the fast discharge in Tyrode solution

As for the mechanism leading to the sudden attainment of the threshold after the first action potential of the run, V_os _could have directly attained the threshold by growing to a value positive to the resting potential (Fig. 4A and 4B). Alternatively, V_os _could have entered the oscillatory zone and initiated a supra-threshold ThV_os_.

**Figure 5:**
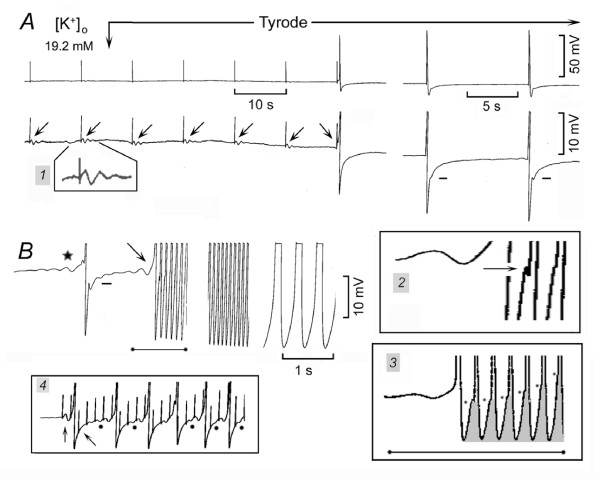
**V_os_, ThV_os _and sudden fast discharge during recovery from high [K^+^]_o _to Tyrode solution**. V_os_, ThV_os _and sudden fast discharge during recovery from high [K^+^]_o _to Tyrode solution. The SAN was quiescent in 19.2 mM [K^+^]_o _and recovery in the Tyrode solution started at the downward arrow and continued for the rest of the figure. In panel A, subthreshold stimuli (15 V, 3 ms; leftward oblique arrows) were applied at 6 min^-1^. Subthreshold ThV_os _is shown at higher gain in inset *1*. In panel B, the star indicates ThV_os _leading to spontaneous discharge and the rightward oblique arrow points to the depolarising slope of a ThV_os _that initiated an AP and fast rhythm. In the inset *2*, the arrow points to the peak of V_os_. The trace indicated by a horizontal line ending with two dots is shown at higher gain in inst *3*, where the dots and the shaded areas emphasize the growth of V_os_. In the inset 4, the subthreshold stimuli initiated ThV_os _at the resting potential (upward arrow), but not during early DD_1 _(leftward oblique arrow). The dots show ThV_os _that began prior to the electrical stimulus.

The contribution of both V_os _and ThV_os _to fast rhythms is illustrated in Fig. 5 during recovery from high [K^+^]_o _to Tyrode solution. In high [K^+^]_o _sub-threshold stimuli at 6 min^-1 ^were followed by sub-threshold ThV_os _(leftward oblique arrows). As shown in inset *1 *at higher gain, ThV_os _consisted of damped depolarizing and hyperpolarizing phases. When Tyrode solution was superfused, the resting potential became more negative and a stimulus-driven ThV_os _(rightward oblique arrow) attained the threshold, thereby initiating an AP. The AP was followed by DD, but not by recognizable V_os _or ThV_os_. The two larger APs at the end of Fig. 5A attained a more negative MDP and were followed by large V_os _(short bars). After these APs were recorded, drive was discontinued.

In Fig. 5B, ThV_os _appeared which reached the threshold an initiated the first AP (star). During the following diastole, the larger and faster depolarizing phase of ThV_os _(arrow) elicited an AP that initiated a sudden fast rhythm (125 min^-1^). The beginning of the fast rhythm is shown at a greater time base in inset *2*. As indicated by the horizontal arrow, the peak of V_os _was still negative to the resting potential and could not possibly have attained the threshold. Instead, with a short delay, V_os _was followed by a depolarizing event that attained the threshold in the potential range of the previous ThV_os_. The trace marked by the line beginning and ending with a dot in Fig. 5B is shown at a greater time base in inset *3*. As V_os _became larger (dots and shaded areas), the delay in the ensuing depolarizing phase became gradually shorter and eventually disappeared. The gradual fusion of V_os _with the apparent depolarizing phase of ThV_os _led to the U-shaped DD (last trace in Fig. 5B). This suggests that the larger V_os  _entered the oscillatory zone and fused with ThV_os _so that the threshold was quickly attained.

The relationship between ThV_os _and voltage (oscillatory zone) is illustrated in inset *4*, where sub-threshold stimuli were applied. The first stimulus caused a ThV_os _with an oscillation above and below the resting potential (vertical upward arrow in inset *4*). The second stimulus increased the depolarizing phase of ThV_os_, which reached the threshold for the upstroke. During the following diastole, the first stimulus (leftward oblique arrow) did not induce a ThV_os_, but the subsequent stimuli fell on a gradually less negative DD and elicited gradually larger ThV_os_. The fifth stimulus enhanced the depolarizing phase of ThV_os _so that it attained the threshold.

As the drive continued, the stimuli during the early DD failed to elicit ThV_os_, but the oscillatory zone shifted to more negative values, as indicated by the fact that some ThV_os _initiated prior to the stimuli at potentials negative to the resting values (dots). Indeed, after the stoppage of the stimuli, spontaneous ThV_os _(still negative to the resting potential) initiated APs (not shown). These results suggest that the fast SAN discharge involves the growth of both V_os _and ThV_os _and that the entry of V_os _into the oscillatory zone is associated with a negative shift of faster and earlier ThV_os_.

**Figure 6:**
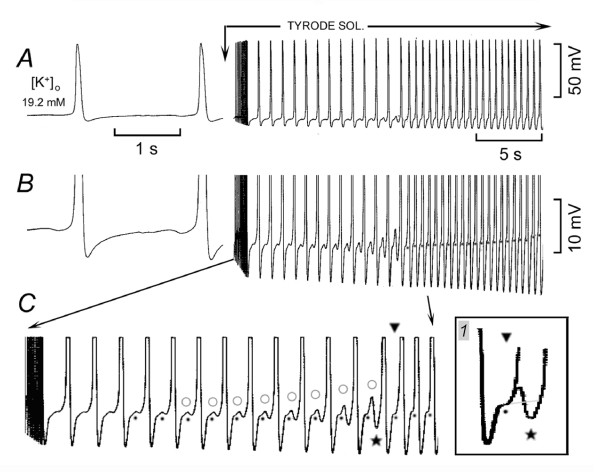
**Growth of V_os _and ThV_os _leading to sudden fast rhythm**. The first part of *A *and *B *panels was recorded in 19.2 mM [K^+^]_o _at normal and higher gain, respectively. Recovery in Tyrode solution began at the downward arrow and continued for the rest of the figure. The section of panel *B *between the two arrows is shown at greater time base in panel *C*. The dots emphasize the growth of V_os _and the circles that of ThV_os_.

The findings illustrated in Fig. 6 directly demonstrate the gradual growth and fusion of V_os _and ThV_os _as well as the negative shift of the oscillatory zone. The SAN was spontaneously active (30 min^-1^) in high [K^+^]_o _and, during the recovery in Tyrode solution, the discharge at first increased gradually and then suddenly (Fig. 6A at normal and *B *at higher gain). As shown at a greater time base in Fig. 6C, the gradual acceleration was associated with a progressive growth of both V_os _(dots) and of the sub-threshold ThV_os_(circles). In addition, the gradually increasing ThV_os _began at more negative potentials (negative shift of the oscillatory zone).

The gradually larger V_os _and ThV_os _eventually fused. At the star, ThV_os _had barely missed the threshold and undershot DD (cf. DD at the beginning of trace 6*C*). During the following diastole (triangle) the depolarizing phase of ThV_os _attained the threshold. The superimposed traces in inset *1 *show that indeed it was ThV_os _(and not V_os_) that reached the threshold, thereby initiating fast discharge. As V_os _kept on increasing (see dots in Fig. 6B), the depolarizing phase of ThV_os _became faster (see last three APs in Fig. 6C), leading to a slight increase in rate of the sudden fast rhythm. Thus, the sudden fast rhythm is different from a gradually increasing discharge in that the fusion of large V_os _and ThV_os _leads to a quick attainment of the threshold for the upstroke.

In *n *= 15, in 16.0 ± 1.7 mM [K^+^]_o_, the SAN was either quiescent or spontaneously active (average rate 34.3 ± 9 min^-1^). During the recovery in Tyrode solution, in quiescent preparations, ThV_os _consistently initiated the spontaneous discharge. The spontaneous discharge increased *gradually *to 163 ± 6.5* min^-1 ^by the time the MDP became more negative by 20.1 ± 1.9 mV. The gradual acceleration was caused by the increase in size and slopes of both V_os _and ThV_os_, the latter occurring progressively sooner during diastole until V_os _and ThV_os _gradually fused.

In *n *= 22, in 14.8 ± 1.6 mM [K^+^]_o_, the SAN was quiescent and during the recovery in Tyrode solution, when the resting potential had repolarized by 4.1 ± 0.4 mV, ThV_os _appeared and consistently initiated APs. A *sudden *fast rhythm initiated either after the very first AP or after a number of slower beats. The rate of the sudden rhythm was 140 ± 8.4* min^-1 ^and it increased to 166 ± 6.2 min^-1 ^(* with respect to 140 min^-1^) by the time the MDP had become more negative by 11.6 ± 1.3 mV.

In *n *= 30, in 16.8 ± 1.2 mM [K^+^]_o_, the SAN was active (either spontaneously active or driven at 6 min^-1^) with an average rate for all tests 19.8 ± 3.7 min^-1^. During the recovery in Tyrode solution, when the diastolic potential had become more negative by 4.2 ± 0.4 mV, a spontaneous or a driven AP initiated a *sudden *fast rhythm at 147 ± 7.3* min^-1^, which gradually increased to 177 ± 4.3 min^-1 ^(* with respect to 147 min^-1^) by the time the MDP had become more negative by 12.5 ± 1.6 mV.

Thus, during the recovery in Tyrode solution (normal [K^+^]_o_), ThV_os _consistently initiated and gradually accelerated SAN discharge. The sudden discharge was due to the summation of large V_os _and early ThV_os _during DD_1_.

### Induction of slow and fast rhythms by overdrive

If adrenergic agents act by increasing [Ca^2+^]_i_, overdriving the SAN should act similarly, since a faster rate increases the intracellular Ca^2+ ^[13]. On this basis, shorter or slower overdrives would be expected to induce a smaller acceleration than longer or faster overdrives. In addition, the greater Ca^2+ ^load with faster overdrive might induce fast rhythms.

**Figure 7:**
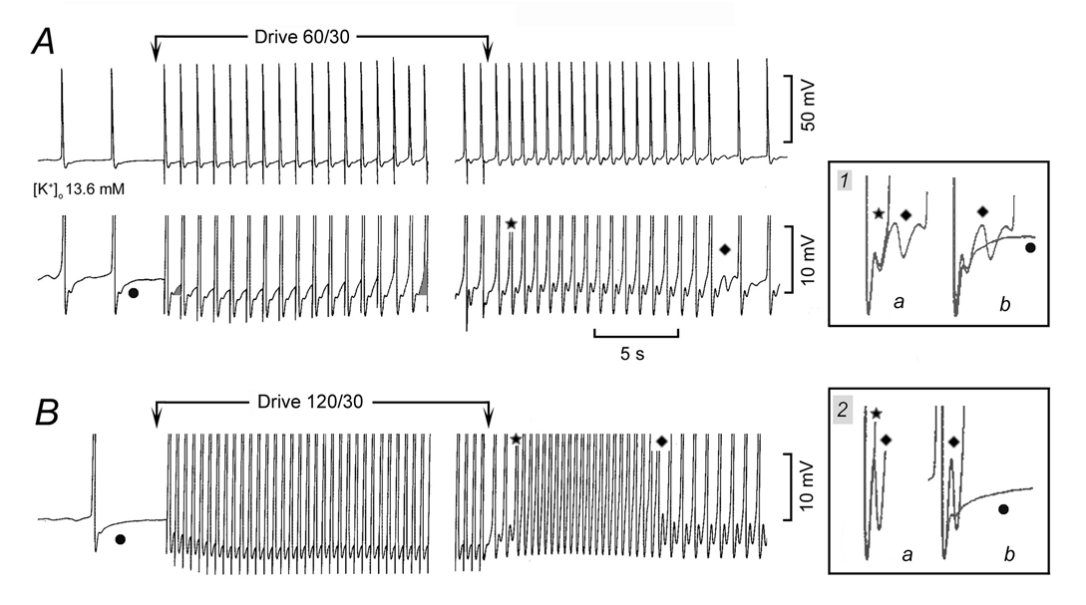
**Induction of fast rhythm by overdrive**. A spontaneously discharging SAN was overdriven at 60 min^-1 ^for 30 s in panel A. In inset *1*, the traces labeled with a star and a rhombus are shown superimposed in *a*, and the traces labeled with a rhombus and a dot are superimposed in *b*. In panel *B*, the SAN was overdriven at 120 min^-1 ^for 30 s. In inset *2*, the traces labeled with a star and a rhombus were superimposed in *a*, and those with a rhombus and a dot in *b*.

In Fig. 7A, in high [K^+^]_o_, the spontaneous rate was 21.4 min^-1^. Overdrive at 60 min^-1 ^progressively increased the size of the depolarizing phase of ThV_os _(cf. shaded areas at beginning and toward end of overdrive), until spontaneous APs preceded the electrical stimuli. On stoppage of drive, larger V_os _and more rapid depolarization of ThV_os _increased the rate of discharge to 85.7 min^-1^. At that rate, the peak of V_os _was followed by ThV_os_. In inset *1*, the superimposed *a *traces show that the subsiding of overdrive excitation was due to ThV_os _missing the threshold. The superimposed *b *traces show that the ThV_os _missing the threshold oscillated above and below the control DD, as ThV_os _usually do.

In Fig. 7B, the spontaneous rate was 5.9 min^-1^. At the end of the 120 min^-1 ^overdrive, after two APs, a sudden fast rhythm began at the rate of 150 min^-1^. The sudden fast rhythm was due to the fusion of the large V_os _and ThV_os_. In inset *2*, the superimposed *a *traces show that the subsiding of the fast rhythm was due to the large ThV_os _missing the threshold. The superimposed *b *traces show that ThV_os _that missed the threshold undershot the control DD. The results support the notion that fast sudden rhythms are brought about by the fusion of larger V_os _and ThV_os _in early diastole due to a larger calcium load.

In *n *= 19, in 13.3 ± 0.9 mM [K^+^]_o_, all preparations but one were quiescent and a slow or short overdrive (average rate 44 min-^1 ^for 43 s) did not induce discharge in 8 tests and caused overdrive excitation in 11 tests at the rate of 23.6 ± 6.7* min^-1^. In no instance, overdrive caused sudden a fast rhythm. In the same preparations, the control rate was 14.3 ± 5.1 min^-1 ^and an overdrive at 95 ± 8 min^-1 ^for 53 ± 7 s, increased the rate to 118 ± 13* min^-1^. In 8/19 of these tests, overdrive induced a sudden fast rhythm at the rate of 154 ± 18 min^-1 ^either immediately or after a gradual acceleration. Therefore, at the same [K^+^]_o_, doubling the driving rate (+113%) consistently induced a much faster overdrive excitation and in 58% of the tests induced a sudden fast rhythm.

### Low [Ca^2+^]_o _and the effects of adrenergic agonists on oscillatory potentials

If adrenergic agonists increase V_os _and ThV_os _by increasing cellular calcium, then low [Ca^2+^]_o _would be expected to antagonize the adrenergic effects on the oscillatory potentials and on the rate increase.

**Figure 8:**
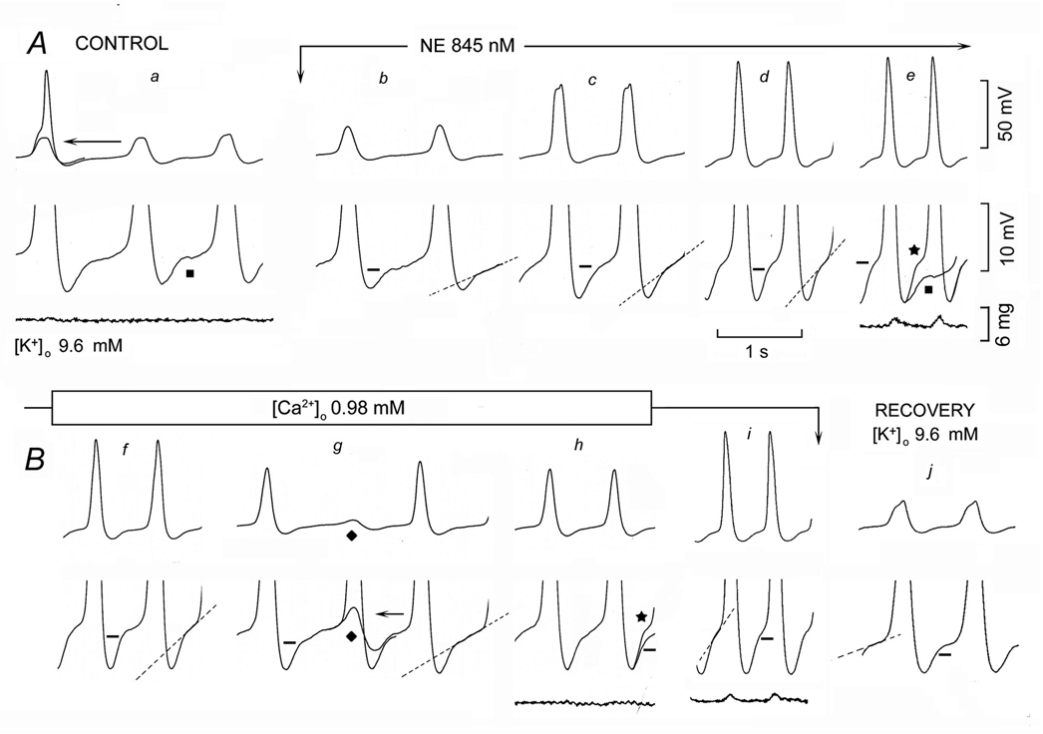
**Low [Ca^2+^]_o _antagonizes the effects of NE**. In panel *a*, the arrow points to the superimposition of the small deflection on the previous AP. NE was administered between the downward arrows. In panel *e*, the control trace labeled with a square was superimposed on the trace marked by a star. [Ca^2+^]_o _was decreased as indicated (panes *f-h*). In panel *g*, the ThV_os _that missed the threshold is labeled with a rhombus and the following AP has been superimposed on it (arrow) in the bottom trace recorded at higher gain. In panel *h*, the trace labeled by a star is the same trace labeled with the same symbol in panel *e*. Panel *i *was recorded in the presence of NE during recovery from low [Ca^2+^]_o_.

In Fig. 8A, in high [K^+^]_o_, the upstroke of the AP was attained with a marked delay and was followed by sub-threshold responses at 28 min^-1 ^(panel *a*). NE increased the amplitude and slope of V_os _(short bars) and of ThV_os _(dash lines) and it induced APs with faster upstrokes and larger magnitude (panels *c-e*). In panel *e*, the superimposition of control diastolic trace (square) on that recorded during NE exposure (star) emphasizes the changes induced by NE on V_os _and ThV_os_. The rate of discharge was faster (303% of control) and the twitch larger.

In Fig. 8B, during NE exposure, lower [Ca^2+^]_o _(-63.7%) gradually decreased V_os _(short bars) and the slope of ThV_os _(dash lines) (panels *f-h*). At the rhombus, ThV_os _missed the threshold, as emphasized by the superimposition of the following AP (arrow) in panel *g*. As shown by the NE trace (star) superimposed on the low [Ca^2+^]_o _trace (panel *h*), lower [Ca^2+^]_o _decreased the magnitude and slope of the oscillatory potentials. The rate was decreased by 30% with respect to NE value and contractions were hardly visible. When [Ca^2+^]_o _was restored to its normal value, the effects of NE on rate and force were restored (panel *i*). The recovery in high [K^+^]_o _is shown in panel *j*.

In *n *= 3, in 13.3 ± 0.9 mM [K^+^]_o_, NE or isoproterenol (mean value 496 ± 272 nM) consistently increased the rate (225%), APa (26%), V_os_ds (194%), V_os_a (83%), DDa (50%) and force (907%). In the presence of adrenergic agonists, lower [Ca^2+^]_o _(1.3 ± 0.4 mM) consistently decreased the rate (-39%), APa (-19%), V_os_ds (-28%), V_os_a (-30%), DDa (-33%) and force (-47%). In a fourth experiment, low [Ca^2+^]_o _induced quiescence [17] and adding NE failed to induce spontaneous activity. In one experiment, during the exposure to isoproterenol, increasing [Ca^2+^]_o _markedly increased rate, DDs and force. Therefore, decreasing [Ca^2+^]_o _counteracted the increased calcium load by adrenergic agonists, thereby reducing their effects on oscillatory potentials and rate. Increasing [Ca^2+^]_o _had the converse effects.

### Cesium effects on dominant and subsidiary SAN pacemakers

Since the oscillatory potentials are integral part of the dominant pacemaker mechanism, the effects of Cs^+ ^(a blocker of I_f_) on the oscillatory potentials were investigated to verify whether I_f _participates in their mechanisms.

**Figure 9:**
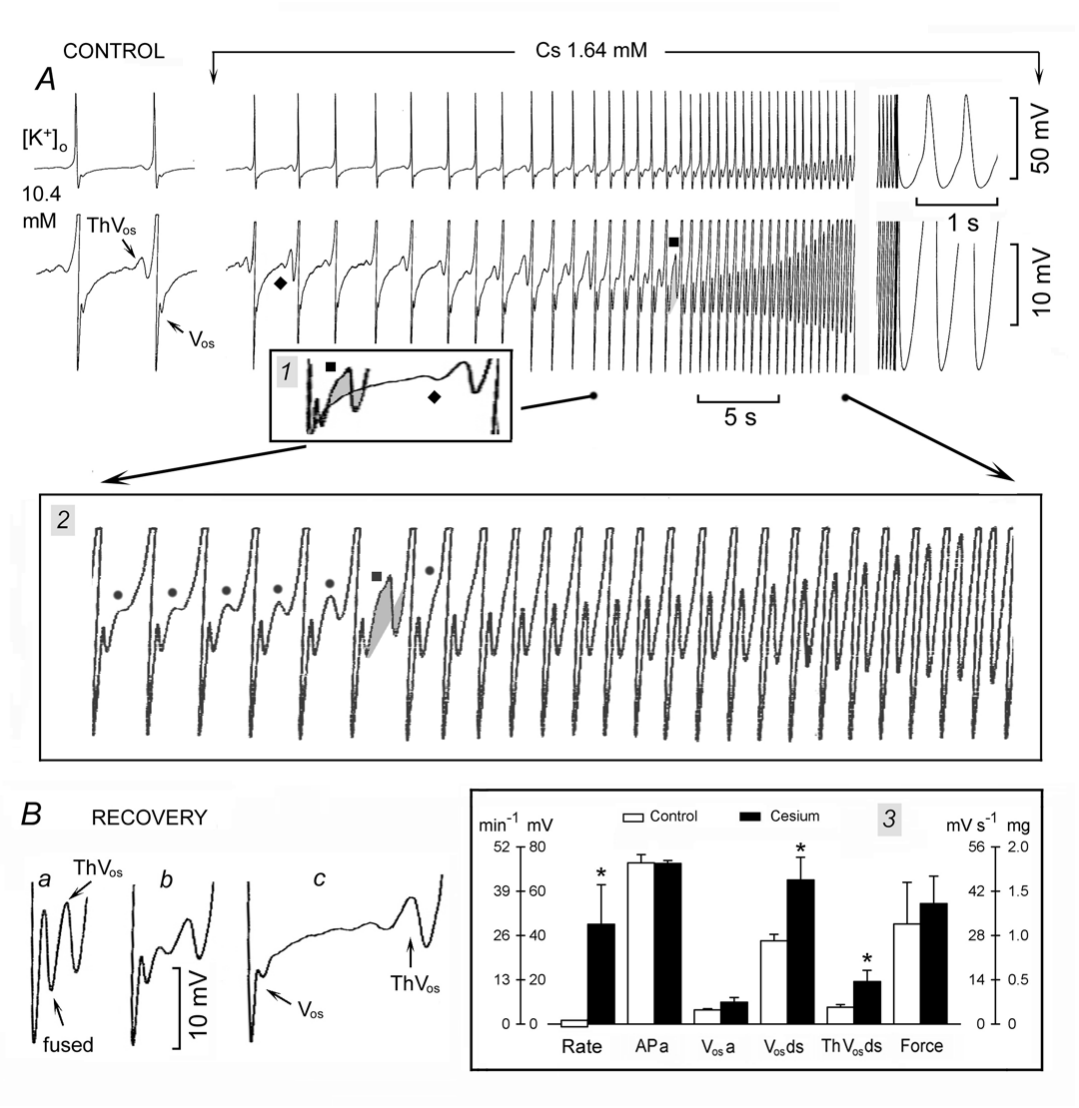
**Cesium does not suppress either V_os _and ThV_os _or SAN discharge**. The APs recorded at normal and higher gain are shown in panel *A *(Control). Cs^+ ^was administered as indicated above panel *A*. In inset *1*, the traces labeled with a square and rhombus are superimposed, with the MDP cut off. Recovery is illustrated in panel *B*. Means and SEM are shown in the inset *3*. Other explanations are in the text and as in legend of Fig. 1.

In Fig. 9A, in a spontaneously active SAN (14 beats min^-1^) in high [K^+^]_o_, the action potential was preceded by ThV_os _and was followed by V_os_, as indicated by the respective arrows. Cs^+ ^did not suppress either the oscillatory potentials or discharge. Instead, Cs^+ ^increased the rate by means of larger and earlier ThV_os _and larger V_os_, as emphasized by the superimposed traces labeled by rhombus and a square without and within inset *1*. The more negative ThV_os _are consistent with a negative shift of the oscillatory zone.

In inset *2*, the progressive changes in V_os _and ThV_os _are shown at greater time base, ThV_os _reaching the threshold by the last dot, as its depolarizing phase became larger and faster. The gradually increasing peak and under-swing of the subsequent diastolic oscillations in inset *2 *suggests that V_os _and the depolarizing phase of ThV_os _were fused (greater peak), the under-swing being caused by the larger hyperpolarizing phase of ThV_os_. Eventually, V_os _and ThV_os _fused in the U-shaped DD (last *A *panel), the rate being 130 beats min^-1 ^(828%).

The converse changes occurred during recovery from Cs^+ ^exposure (Fig. 9B). When the first fused diastolic oscillation (upward oblique arrow) missed the threshold, a large ThV_os _was present (downward oblique arrow), although only a subsequent one initiated an AP (trace *a*). Later on, V_os _was smaller and clearly separated from the subsequent smaller ThV_os _(trace *b*). The complete separation between V_os _and ThV_os _is shown in trace *c*, where DD is interposed between the two oscillatory potentials. The depolarizing and hyperpolarizing slopes of ThV_os _gradually became slower and the oscillatory zone returned to less negative values.

In *n *= 5, (Fig. 9, inset *3*), in SAN quiescent in 13.3 ± 0.4 mM [K^+^]_o_, 1.5 ± 0.2 mM Cs^+ ^induced spontaneous discharge at 29* min^-1 ^and induced the following changes: APa -0.002%, V_os_a 53%, V_os_ds 73%*, ThV_os_ds 132%* and force 20% (APd -2.9%, DDa 23%, not shown).

In *n *= 14, in SAN active in 13.6 ± 0.8 mM [K^+^]_o_, 1.7 ± 0.1 mM Cs^+ ^induced the following changes: rate 97%*, APa -0.002%, APd 0.006%, V_os_ds 116%, V_os_a 8.6%, ThV_os_ds 330%*, DDa -0.01% and force 48%. Therefore, Cs^+ ^did not abolish V_os_, ThV_os _or DD, indicating that I_f _does not play a role in the mechanisms underlying these events. This agrees with findings that in SAN Cs^+ ^blocks I_f_, but not spontaneous discharge in Tyrode solution [19,25] or in high [K^+^]_o _[15].

The more negative DD of SAN subsidiary pacemakers appears to be due to the activation of I_f _[19] and therefore Cs^+ ^would be expected to have on subsidiary pacemakers in Tyrode solution effects different from those on dominant pacemakers (see [26,27]). In Tyrode solution (*n *= 8), 4.4 ± 1.2 mM Cs^+ ^induced the following changes: rate -18.7%*, APa 1.5%, APd 93%*, DDa -81%*, DDs -87%* and force 0.0%. The increase in APd was due to a slowing of the late phase 3 repolarization (the "tail") whose amplitude was 31.8 ± 2.9* mV and slope was 311 ± 18.9* mV s^-1 ^(see [26]).

### Cesium and the positive chronotropic effect of norepinephrine

Even if SAN oscillatory potentials do not appear to involve I_f_, still the positive chronotropic effect of NE could be mediated by a positive shift of the activation curve of I_f _[2,21]. If it were so, then Cs^+ ^should antagonize the acceleratory action of NE by blocking I_f_, thereby making its positive shift irrelevant. This was tested by giving Cs^+ ^first (to block I_f_) and then by adding NE.

**Figure 10:**
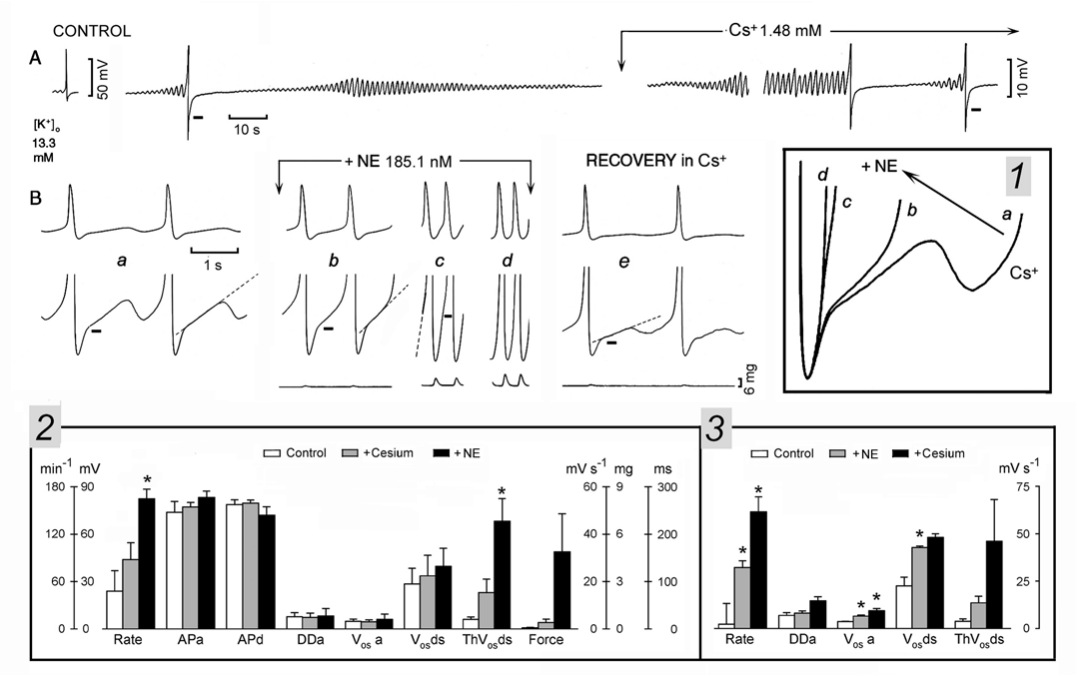
**Norepinephrine increases the rate of discharge in the presence of Cs^+^**. In high [K^+^]_o_, Cs^+ ^administration was initiated at the downward arrow and continued for the rest of the figure. NE was added to the Cs^+ ^solution as indicated above traces *b*, *c *and *d*. Recovery in Cs^+ ^solution (no NE) is shown in trace *e*. Means and SEM are shown in the inset *2 *(Control, +cesium, +NE) and *3 *(Control, +NE, +cesium). Other explanations are in the text and as in legend of Fig. 1.

In Fig. 10A control, a spontaneous AP is shown in the first panel and the lower part of the same AP (followed by diamond pattern and quiescence) is shown in the second panel. Cs^+ ^administration made ThV_os _re-appear, gradually increase in size and re-initiate spontaneous discharge. Typically, each AP was followed by V_os _and by the temporary suppression of ThV_os_. The latter reappeared when DD re-entered the oscillatory zone. Discharge gradually increased until a steady rhythm was present (29 beats min^-1^, Fig. 10B, panel *a*). Thus, (in contrast to early afterdepolarizations) the pre-potential ThV_os _can occur at the resting potential in the absence of APs and it is temporarily suppressed by an action potential.

When NE was added to the Cs^+ ^solution, V_os _amplitude (short bars) and ThV_os _slope (dash lines) increased and attained the threshold with each ThV_os _(64.8 beats min^-1^, panel *b*). The greater V_os _and much steeper slope of ThV_os _further increased the rate (130 beats min^-1^, panel *c*) and led to U-shaped DD (176 min^-1^, panel *d*). Contractile force increased during NE exposure by 633%. In inset *1*, the traces (superimposed by the MDP) show that the marked increase in rate from trace *a *(Cs^+^) to trace *b *(+NE) was due to NE-induced steepening of depolarizing phase of ThV_os_, which allowed it to initiate an AP. The subsequent increase in rate involved both an increase in V_os _amplitude and a much steeper slope of ThV_os _(trace *c*), until V_os _and ThV_os _became fused and no longer distinguishable (trace *d*). During the recovery from NE in the Cs^+ ^solution, the rate slowed through the decrease of V_os _and ThV_os _amplitude (panel *e*).

In inset *2 *(*n *= 4), in SAN active in 13.6 ± 1 mM [K^+^]_o_, 1.6 ± 0.2 mM Cs^+ ^induced the following changes: rate 84%, APa 4.6%, APd 1.1%, DDa -6.5%, V_os_a -7.7%, V_os_ds 17.9%, ThV_os_ds 280% and force 471%. Adding 351 ± 58 nM NE induced the following changes with respect to the Cs^+ ^values: rate 87.2%*, APa 7.8% APd -9.6%, DDa 15.7%, V_os_a 36.9%, V_os_ds 17.8%, ThV_os_ds 56.6%, and force 1118.5%.

If Cs^+ ^did not prevent the acceleratory action of NE, NE did not prevent a further increase in rate of discharge by Cs^+^, indicating that Cs^+ ^has the same effect in the absence (basal rhythm) and in the presence of NE. The effects of Cs^+ ^were reversible in NE solution, showing that the further acceleration during Cs^+ ^exposure was not due to a still increasing positive chronotropic effect of NE. In *n *= 2 (inset 3), in SAN active in 13.6 ± 1 mM [K^+^]_o_, NE (279 ± 95 nM) induced the following changes: rate 1378%*, DDa 18.1%, V_os_a 82.3%*, V_os_ds 90%*, ThV_os_ds 260% (APa 6.1%, APd -11.5%, not shown). Adding Cs^+ ^(1.96 ± 8 mM) induced the following changes with respect to the NE values: rate 91.6%*, DDa 79.4%, V_os_a 45%*, V_os_ds 12.4% and ThV_os_ds 241% (APa -0.08%, APd 4.3%, not shown).

### Barium and the oscillatory potentials

The block of I_f _by Cs^+ ^did not abolish the oscillatory potentials. Instead, Cs^+ ^actually *increased *the rate of discharge, certainly not through its block of I_f _and possibly through a decrease in K^+ ^conductance. If so, barium should also increase the rate of discharge, since Ba^2+ ^blocks K^+ ^currents, but not I_f _[28].

**Figure 11:**
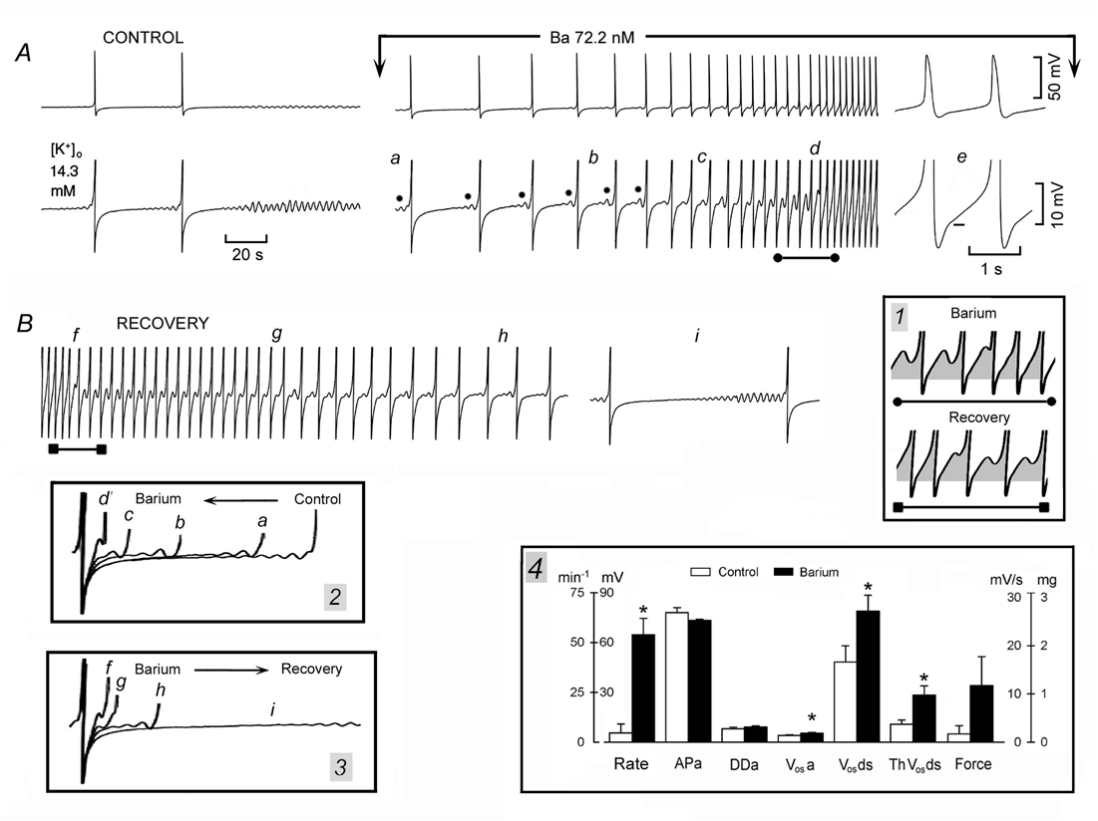
**Barium increases V_os _and ThV_os _slope and amplitude, and rate of discharge**. In panel A, APs in high [K^+^]_o _are shown at normal and higher gain. Ba^2+ ^was administered as indicated above the top trace. The dots label ThV_os _occurring gradually sooner during DD_2_. In panel B, the recovery from Ba^2+ ^exposure is shown. Inset *1 *shows the changes in slope and amplitude of ThV_os _during (top trace) and after barium exposure (bottom trace). In inset *2 *and 3, the arrows indicate the direction of change during and after barium exposure, respectively. Means and SEM are shown in inset *4*. Other explanations are in the text and as in legend of Fig. 1.

In Fig. 11A, in SAN superfused in high [K^+^]_o_, the control panel shows two APs, followed by the diamond pattern and quiescence. Exposure to a low concentration of Ba^2+ ^made ThV_os _re-appear and initiate discharge that quickly accelerated due to earlier and larger ThV_os _(dots) as well as larger DD_2 _(traces *a-d*). As shown in panel *e*, after the peak of V_os _(short bar), the depolarizing slope of ThV_os _reached the threshold for the upstroke. The section of trace *d *labeled with the line terminating with dots and displayed at a greater time base in inset *1 *(top trace) shows that Ba^2+ ^quickly increased the depolarizing phase of sub-threshold ThV_os_so that it consistently reached the threshold (shaded areas).

In Fig. 11B, during the recovery from Ba^2+ ^exposure, the amplitude of the depolarizing phase of ThV_os _gradually decreased and intermittently missed the threshold. ThV_os _began gradually later during DD (traces *f-h*). Eventually, ThV_os _appeared only during the late DD_2 _(trace *i*) and quiescence followed shortly thereafter. The section of trace *f *labeled with a line terminating with squares and displayed at greater time base in inset *1 *(bottom trace) shows the converse changes with respect to the top trace. Namely, the depolarizing phase of ThV_os _missed the threshold and quickly decreased in size (shaded areas) with a consequent decrease in rate.

In inset *2*, the superimposed traces show that Ba^2+ ^increased the amplitude of DD and caused ThV_os _to appear gradually sooner. Conversely, during the recovery from Ba^2+ ^(inset *3*), ThV_os _became smaller and started later during a more negative DD_2_.

In inset *4*, *n *= 6, in SAN mostly quiescent in 15.1 ± 0.9 mM, [K^+^]_o_, Ba^2+ ^(89.6 ± 13.7 nM) depolarized the resting membrane by 4.2 ± 0.9 mV and induced the following changes: rate 1078%*, APa -5.8%, DDa 13.5%, V_os_a 36.2%*, V_os_ds 63.4%*, ThV_os_ds 162.1%*, and force 588% (APd 20.4%*, not shown). Thus, in high [K^+^]_o_, Ba^2+ ^(a blocker of K^+ ^conductance) increased the rate through an increase in amplitude of V_os_, ThV_os _and DD, as Cs^+ ^does.

## Discussion

From the results obtained, we conclude that adrenergic activation increases the SAN rate by increasing the pacemaking components DD_1_, V_os _and ThV_os_. Their fusion by NE in high [K^+^]_o _or during the recovery from high [K^+^]_o _to Tyrode solution leads to the U-shaped DD, typical of dominant pacemakers. In quiescent SAN, adrenergic agonists initiate spontaneous discharge by decreasing the resting potential, thereby inducing ThV_os_. Gradually earlier and larger ThV_os _are mainly responsible for a gradual acceleration in rate, and the sudden fusion of large V_os _and ThV_os _for the induction of fast rhythms.

Adrenergic agonists appear to act by increasing [Ca^2+^]_i_, since other procedures known to increase cellular calcium (e.g., lowering high [K^+^]_o _and overdrive) also increased SAN discharge by increasing the size and slope of V_os _and ThV_os _and by shifting the oscillatory zone in a negative direction. Conversely, low [Ca^2+^]_o _antagonizes the effects of adrenergic agonists. A larger calcium load by NE, lower [K^+^]_o _and faster overdrives facilitates the abrupt onset of fast rhythms.

Even though it has been reported that adrenergic stimulation can change the gating and amplitude of I_f _in SA nodal cells (see [2]), the experiments with Cs^+ ^show that I_f _modulation does not contribute to the oscillatory potentials involved in dominant discharge or to their modifications by adrenergic agonists. The *increase *in rate of discharge by Cs^+ ^appears to involve a decrease in potassium conductance, since similar results were obtained with Ba^2+^. The different effects of Cs^+ ^on *dominant *pacemakers in high [K^+^]_o _and in *subsidiary *pacemakers in Tyrode solution suggest to a role of I_f _in the more negative subsidiary diastolic depolarization.

### Mechanisms of initiation of pacemaker activity by adrenergic agonists

For NE to decrease the resting potential, an increase in a net inward current is required. Several currents do not appear to be involved in NE-induced depolarization. The block of I_f _by Cs^+ ^did not suppress the depolarization in the absence (present results) and in the presence of nifedipine [29]. I_CaT _is not increased by adrenergic agonists [7,9] and, at the holding potential of -40 mV, I_CaT _[7] and voltage-dependent Na^+ ^channels would be inactivated. A decrease or positive shift of I_K _is ruled out, since isoproterenol increases the amplitude of I_K _and causes a negative shift of its activation curve [10]. A decrease in the outward Na^+^-K^+ ^pump current is also unlikely to be involved, since NE stimulates the Na^+^-K^+ ^pump activity [30,31]. Therefore, both an increase in I_K _and in Na^+^-K^+ ^pump activity would hyperpolarize (not depolarize) the resting membrane.

Adrenergic activation decreased the resting potential in guinea pig SAN arrested by 10 μM nifedipine [29], demonstrating that the depolarization occurs after the block of I_CaL_. Exogenous NE caused depolarization by releasing calcium from SR stores through the activation of metabolic pathways [29]. This intracellular release of calcium activates an inward I_Na-Ca_, which depolarizes the resting membrane.

In the present experiments, in the absence of nifedipine, adrenergic activation would be expected to increase also I_Ca_. Thus, adrenergic agonists lead to a cAMP-mediated PKA-dependent phosphorylation not only of the ryanodine receptors and of phospholamban, but also of the L-type Ca^2+ ^channels [3,12]. A contribution of sub-threshold I_Ca _to the decrease of the resting potential is made likely by the fact that the resting potential of the SAN (~-40 mV) is fairly close to the threshold for I_Ca _activation [21]. In high [K^+^]_o_, nifedipine stopped SAN discharge by preventing the attainment of the threshold [15]. In SAN quiescent in high [K^+^]_o_, high [Ca^2+^]_o _decreased the resting potential, thereby leading to the appearance of ThV_os _and of discharge [16].

During the decrease of the resting potential, sub-threshold Ca^2+ ^entry induced by NE or high [Ca^2+^]_o _could also release Ca^2+ ^from the SR. However, the primary event would be the enhanced Ca^2+ ^influx through the surface membrane, as in the case of the latent atrial pacemakers [11] and of an inward calcium component elicited either by a small depolarizing step during late DD [32] or at -40 mV during ramps from -60 to -20 mV [21].

The decrease in resting potential, by entering the oscillatory zone, allowed ThV_os _to appear and initiate discharge (Fig. 1; Fig. 3, panel *f*). Therefore, not the depolarization per se, but the onset of ThV_os _was responsible for the initiation of discharge. Also, in quiescent Purkinje fibers, NE decreased the resting potential, induced ThV_os _and initiated spontaneous discharge [33]. However, in Purkinje fibers, not only the resting potential is much more negative to the threshold for I_Ca_, but the depolarizing phase of ThV_os _is related to a slowly inactivating sodium current (I_Na3_) which is activated in the oscillatory zone of Purkinje fibers (~-60 to -50 mV) [34].

### Mechanisms of gradual increase in rate by adrenergic agonists

The gradual increase in SAN discharge by adrenergic agonists involves (in addition to ThV_os_) two additional mechanisms, namely, DD_1 _and V_os_. Both events obligatorily follow the action potential, in contrast to ThV_os_.

*As for DD*_1_, in the presence of background sodium current, the decay of I_K _leads to DD (e.g., see [4,10,19,32]). NE increases the magnitude of I_K_, shifts its activation curve in a negative direction [10] and speeds up its rate of deactivation [10,35], thereby accounting for the shortening of the AP, the increase in MDP and the steepening of a larger DD_1_.

With a resting potential of -40 mV, the already deactivated I_f _can not decrease during the AP to account for the subsequent undershoot to the MDP [36]. Further, I_f _activates too slowly at the MDP to account for DD (see [4]). In any case, the undershoot was not abolished by Cs^+^, as it would be expected if it were due to the deactivation of I_f _during the AP. Instead, the undershoot persisted, because Cs^+ ^does not block I_K_[37].

*As for V*_os_, NE-induced phosphorylation of L-type Ca^2+ ^channels increases I_Ca _[6] and the Ca^2+ ^transient [8,14]. At the same time, the phosphorylation of phospholamban and of the ryanodine receptors increases Ca^2+ ^uptake into as well as release from the SR (see [38]). The greater SR release of calcium during late diastole results in larger I_Na-Ca _[8,11] and in a faster and larger V_os_. In turn, V_os _steepens DD_1 _and brings it closer to the oscillatory zone.

Discharge is enhanced also by the negative shift of the oscillatory zone (the threshold for ThV_os _becomes more negative). The increase in [Ca^2+^]_i _could shift the oscillatory zone in a negative direction for the same reason that an increase in [Ca^2+^]_o _shifts the threshold for the fast sodium current I_Na _in a positive direction, namely, by screening surface membrane negative charges.

Also in Purkinje fibers, NE increases the amplitude of V_os _induced by other interventions [39-41]. In fact, interventions that increase cellular Ca^2+ ^(low [K^+^]_o_, high [Ca^2+^]_o_, repetitive depolarizing steps, strophanthidin as well as NE) markedly increased the slope and amplitude of I_os _(the current underlying V_os_) and therefore also the slope of the pacemaker current upon which I_os _is superimposed [42].

The NE-induced gradual increase in rate was predominantly due to the changes in ThV_os_, but the amplitude of V_os _and DD also increased. Eventually, the fusion of the enhanced DD_1_, V_os _and ThV_os _resulted in a steeper slope of the U-shaped DD. In Tyrode solution, adrenergic agonists steepen the slope of the U-shaped DD through the same changes, since an increased calcium load increases V_os _and ThV_os _when visible in the SAN (e.g., [43]) as well as in Purkinje fibers (e.g., [22,41]). Therefore, the U-shaped DD due to the fusion of larger V_os_, ThV_os _and DD_1 _is present not only when norepinephrine is administered in high [K^+^]_o_, but in other conditions that also increase intracellular calcium, such as a suitable reduction in high [K^+^]_o_, after fast overdrive and during recovery from high [K^+^]_o _to Tyrode solution.

### Mechanisms of sudden onset of fast rhythms by adrenergic agonists

As for the sudden onset of fast rhythms, the mechanism of initiation required at least one AP, pointing to the essential role of V_os_. However, the simultaneous growth of ThV_os _and the negative shift of the oscillatory zone contributed to the initiation and maintenance of sudden fast rhythms. Both V_os _and ThV_os _grew prior to the onset of the sudden fast rhythm, but (when V_os _and ThV_os _fused suddenly) it was ThV_os _that attained the threshold for the upstroke (see Fig. 6).

The contribution of both V_os _and ThV_os _to the fast discharge is also indicated by the large oscillations unmasked by the sudden cessation of the fast rhythm. Damped oscillations are seen also with large V_os_(e.g., [40]) and I_os _(e.g, [44]), but the contribution of fused ThV_os _is suggested by the fact that the largest hyperpolarizing phase of the oscillations undershot DD: ThV_os _does that [17], but not I_os _[42].

Since an increase in rate *per se *enhances calcium loading, the further increase in V_os _and ThV_os _accounts for the increase in rate during the fast rhythm, as also suggested by the large oscillations that followed its sudden cessation. Therefore, the initiation of sudden fast rhythms depends on a sudden fusion of larger V_os _and ThV_os_, whereas the gradual acceleration of the rate is due to the increment in the oscillatory potentials, the gradually earlier onset of ThV_os _and more negative oscillatory zone.

The induction of sudden fast rhythms by fast (but not slow) overdrive suggests that a sudden increase in rate is due to a greater Ca^2+ ^load. Thus, a slow/short overdrive induced a ThV_os_-dependent slow rhythm, whereas fast/long drives often induced a sudden fast rhythm. The fast rhythms in high [K^+^]_o _reflect the mechanism underlying the SAN fast discharge in Tyrode solution, as demonstrated here in the guinea pig by the unmasking of diastolic oscillations by high [K^+^]_o _and their progressive growth and fusion on return to Tyrode solution.

The induction or acceleration of discharge by overdrive is not unique to the SAN, since it was first described and labeled "overdrive excitation" in Purkinje fibers overdriven in the presence of NE [45] (later on, overdrive excitation was also labeled "triggered activity").

*In vivo*, in animals with *chronic *complete atrio-ventricular (A-V) block, stimulation of the stellate ganglion [46] or of the splanchnic nerves to the adrenal medulla [47] induced a gradual increase in idioventricular rate. In animals with *acute *A-V block, the operative and postoperative stress is associated with an increased plasma level of catecholamines. When calcium load was increased by left stellate ganglion stimulation, NE administration, Ca^2+ ^infusion, overdrive could induce ventricular tachycardias, which initiated and stopped abruptly [24]. In Purkinje fibers superfused *in vitro*, when the Ca^2+ ^load was increased by lower [K^+^]_o_, high [Ca^2+^]_o_, low [Na^+^]_o_or NE administration, overdrive induced V_os _and ThV_os_, and fast discharge [22,41].

Therefore, *in vitro *as well as *in vivo*, in cardiac pacemakers sympathetic enhancement can induce both a gradual or sudden change in rate. The role of an adrenergically-increased Ca^2+ ^load in increasing SAN rate is shown by similar results obtained with procedures that increase [Ca^2+^]_i_, such as decreasing high [K^+^]_o_, recovery in Tyrode solution and faster or longer overdrives. Reciprocally, a lower [Ca^2+^]_o _antagonized the effects of the adrenergic agonists. Because catecholamines increase cellular calcium whereas high [K^+^]_o_ decreases it, it is not surprising that the resistance of SAN discharge to high [K^+^]_o _in part depends on the degree of sympathetic activation [48].

### I_f_, oscillatory potentials and increase in rate by norepinephrine

The experiments with Cs^+ ^addressed three separate issues. The first issue was the role of the hyperpolarization-activated I_f _in the oscillatory potentials. If the hyperpolarization-activated I_f _were responsible for the oscillatory potentials, cesium (by blocking I_f_) should have suppressed them and stopped SAN discharge under basal conditions. This did not occur, indicating that I_f _is not involved in the mechanism of the oscillatory potentials.

The second issue was related to the suggestion that adrenergic agents increase the discharge of SAN by shifting the activation curve of I_f _to less negative potentials [2,21]. If so, then cesium (by blocking I_f_) should have prevented its positive shift by NE and, therefore, would have abolished the consequent increase in rate. Since NE increased SAN discharge by enhancing the oscillatory potentials also in the presence of cesium, the result indicates that the positive chronotropic effect of NE does not involve an action of I_f _on the oscillatory potentials. This agrees with the finding that epinephrine increases the rate of discharge of SAN in the presence of a demonstrated block of I_f _by Cs^+ ^[6]. Also, in canine SAN, blockade of I_f _with ZD 7288 decreased SAN rate by 8.3%, but neither prevented the isoproterenol-induced increase in rate nor the late diastolic Ca^2+ ^elevation [49].

The third issue was that the mechanism by which cesium *increased *the basal rate of discharge. This mechanism could not be the block of I_f _and might be related to a decrease in potassium conductance. Indeed, in ventricular myocytes [50] and in Purkinje fibers (see [5]), high [K^+^]_o _increases I_K1 _conductance, although I_K1 _is far less expressed in SAN dominant pacemaker cells than in ventricular or Purkinje cells. This hypothesis was tested by using barium, that (like cesium) decreases potassium conductance, but (unlike cesium) does not block I_f _[28]. The results show that the decrease in potassium conductance appears responsible for the increase in rate by cesium or barium.

## Conclusion

The major action of adrenergic agonists appears related to the greater calcium load. The greater Ca^2+ ^influx and the greater Ca^2+ ^release from SR during systole increase contractile force. The greater release of Ca^2+ ^during diastole in SAN dominant pacemakers increases I_Na-Ca _and therefore amplitude and slope of V_os_. An increase in [Ca^2+^]_i _also increases the slope and magnitude of ThV_os _and shifts the threshold to more negative values by shifting the oscillatory zone. The overall changes result in a faster rate of discharge, faster conduction and stronger contraction, which, in turn, lead to a larger cardiac output. Therefore, in all cells adrenergic control of the heart involves an increase in [Ca^2+^]_i_. In dominant pacemaker cells, such a Ca^2+ ^increase enhances the factors involved in SAN discharge (DD, V_os _and ThV_os_).

As for clinical implications, in a healthy person, the activation of the sympathetic system, would be expected to lead to the gradual increase in heart rate, which contributes to an increase in cardiac output (e.g., exercise). In a patient in cardiac arrest, intracardiac injection of NE may initiates spontaneous activity by eliciting ThV_os _in Purkinje fibers. If the SR of the cardiac cells is already calcium overloaded by disease (see [51]), then an increased adrenergic activation may lead to abrupt tachycardias by allowing the fused V_os _and ThV_os _in pacemaker tissues or large V_os _in myocardial tissues to suddenly attain the threshold for the upstroke.

Because V_os _and ThV_os _size as well as the negative shift of the oscillatory zone are a function of [Ca^2+^]_i_, a sudden tachycardia implies a larger Ca^2+ ^overload and/or a diminished ability of cardiac cells to cope with such a load. This may result from an increased Ca^2+ ^load, e.g., a prolonged adrenergic activation, a persistent increase in rate (e.g., cardiac failure), a reduced Ca^2+ ^extrusion due to a reduced Na^+^-K^+ ^pump activity (e.g., ischemia or toxic concentrations of cardiac glycosides) (see [51]) or to a decrease in [K^+^]_o_(hypokalemia).

## Competing interests

The authors declare that they have no competing interests.

## Authors' contributions

This work was a collaborative endeavor in which all authors were involved under the guidance, coordination and major participation of MV in all aspects of the research process in succession (design, experiments, discussion of results, measurements of results, preparation of figures, statistical analysis, and drafting of the manuscript). All experimental work was carried out in the Department of Physiology and Pharmacology, State University of New York, Downstate Medical Center, Brooklyn, NY 11203, USA. All authors read and approved the final manuscript.
